# RETRACTION: The Novel Role of Circular RNA ST3GAL6 on Blocking Gastric Cancer Malignant Behaviours Through Autophagy Regulated by the FOXP2/MET/Mtor Axis

**DOI:** 10.1002/ctm2.70025

**Published:** 2024-09-24

## Abstract

**RETRACTION**: P. Xu, X. Zhang, J. Cao, J. Yang, Z. Chen, W. Wang, S. Wang, L. Zhang, L. Xie, L. Fang, Y. Xia, Z. Xuan, J. Lv, H. Xu, and Z. Xu, “The Novel Role of Circular RNA ST3GAL6 on Blocking Gastric Cancer Malignant Behaviours Through Autophagy Regulated by the FOXP2/MET/Mtor Axis,” *Clinical and Translational Medicine* 12, no.1 (2022): e707, https://doi.org/10.1002/ctm2.707.

The above article, published online on 21 January 2022 in Wiley Online Library (wileyonlinelibrary.com), has been retracted by agreement between the journal Editor‐in‐Chief, Xiangdong Wang; the Shanghai Institute of Clinical Bioinformatics; and John Wiley & Sons Australia, Ltd. The retraction has been agreed upon following an investigation into concerns raised by a third party, which revealed inappropriate duplications of image panels within the article (Figures 6H and 7J) and between this article (Figures 2D, 7B, 7K, 8E and 8J) and another article previously published elsewhere by an overlapping group of authors in a different scientific context. The authors were unable to provide a satisfactory explanation and the raw data they supplied could not explain the identified issues. In addition, the cell line SGC‐7901 used in this study has been reported as contaminated with HeLa cells, making it a problematic model for gastric cancer [1,2]. Given the extent of the identified issues, the editors have lost confidence in the data presented and the article's conclusions can no longer be considered reliable.

References

[1] F. Ye, C. Chen, J. Qin, J. Liu, and C. Zheng, “Genetic Profiling Reveals an Alarming Rate of Cross‐Contamination Among Human Cell Lines Used in China,” *The FASEB Journal* 29, no. 10 (2015):4268–4272, https://doi.org/10.1096/fj.14‐266718.

[2] X. Bian, Z. Yang, H. Feng, H. Sun, and Y. Liu, “A Combination of Species Identification and STR Profiling Identifies Cross‐contaminated Cells from 482 Human Tumor Cell Lines,” *Scientific Reports* 7, no. 1 (2017):9774, https://doi.org/10.1038/s41598‐017‐09660‐w.

**RETRACTION**: P. Xu, X. Zhang, J. Cao, J. Yang, Z. Chen, W. Wang, S. Wang, L. Zhang, L. Xie, L. Fang, Y. Xia, Z. Xuan, J. Lv, H. Xu, and Z. Xu, “The Novel Role of Circular RNA ST3GAL6 on Blocking Gastric Cancer Malignant Behaviours Through Autophagy Regulated by the FOXP2/MET/Mtor Axis,” *Clinical and Translational Medicine* 12, no.1 (2022): e707, https://doi.org/10.1002/ctm2.707.

The above article, published online on 21 January 2022 in Wiley Online Library (wileyonlinelibrary.com), has been retracted by agreement between the journal Editor‐in‐Chief, Xiangdong Wang; the Shanghai Institute of Clinical Bioinformatics; and John Wiley & Sons Australia, Ltd. The retraction has been agreed upon following an investigation into concerns raised by a third party, which revealed inappropriate duplications of image panels within the article (Figures 6H and 7J) and between this article (Figures 2D, 7B, 7K, 8E and 8J) and another article previously published elsewhere by an overlapping group of authors in a different scientific context. The authors were unable to provide a satisfactory explanation and the raw data they supplied could not explain the identified issues. In addition, the cell line SGC‐7901 used in this study has been reported as contaminated with HeLa cells, making it a problematic model for gastric cancer [1,2]. Given the extent of the identified issues, the editors have lost confidence in the data presented and the article's conclusions can no longer be considered reliable.

**References**

[1] F. Ye, C. Chen, J. Qin, J. Liu, and C. Zheng, “Genetic Profiling Reveals an Alarming Rate of Cross‐Contamination Among Human Cell Lines Used in China,” *The FASEB Journal* 29, no. 10 (2015):4268–4272, https://doi.org/10.1096/fj.14‐266718.

[2] X. Bian, Z. Yang, H. Feng, H. Sun, and Y. Liu, “A Combination of Species Identification and STR Profiling Identifies Cross‐contaminated Cells from 482 Human Tumor Cell Lines,” *Scientific Reports* 7, no. 1 (2017):9774, https://doi.org/10.1038/s41598‐017‐09660‐w.

